# Physical Stability and Molecular Mobility of Resveratrol in a Polyvinylpyrrolidone Matrix

**DOI:** 10.3390/molecules30091909

**Published:** 2025-04-25

**Authors:** Aleksandra Pajzderska, Miguel Angel González, Marcin Jarek, Jadwiga Mielcarek, Jan Wąsicki

**Affiliations:** 1 Faculty of Physics, Adam Mickiewicz University, Uniwersytetu Poznanskiego 2, 61-614 Poznan, Poland; 2 Institute Laue Langevin, 71 Avenue des Martyrs, 38042 Grenoble, France; gonzalezm@ill.fr; 3 NanoBioMedical Centre, Adam Mickiewicz University, Wszechnicy Piastowskiej 3, 61-614 Poznan, Poland; marcin.jarek@amu.edu.pl; 4 Department of Inorganic and Analytical Chemistry, University of Medical Sciences, Grunwaldzka 6, 60-780 Poznan, Poland; jmielcar@ump.edu.pl

**Keywords:** polyvinylpyrrolidone, resveratrol, physical stability, molecular mobility, relaxometry, time-domain NMR

## Abstract

The physical stability, molecular mobility, and appearance of nanocrystalline resveratrol in a polyvinylpyrrolidone (PVP) matrix were investigated. Two formulations with resveratrol loadings of 30% and 50% were prepared and characterized using powder X-ray diffraction (PXRD) and time-domain nuclear magnetic resonance (TD-NMR). Samples were studied over time (up to 300 days post-preparation), across temperatures (80–300 K), and under varying humidity conditions (0% and 75% relative humidity). The results demonstrate that the 30% resveratrol–PVP sample is a homogeneous amorphous solid dispersion (ASD), while the 50% resveratrol–PVP sample contained resveratrol nanocrystals measuring about 40 nm. NMR measurements and molecular dynamics (MD) simulations revealed that incorporation of resveratrol into the polymer matrix modifies the system’s dynamics and mobility compared to the pure PVP polymer. Additionally, MD simulations analyzed the hydrogen bonding network within the system, providing insights for a better understanding of the physical stability of the ASD under different conditions.

## 1. Introduction

Resveratrol (trans-3,5,4′-trihydroxystilbene, C_14_H_12_O_3_, hereafter RSV) belongs to the stilbenoids, a group of naturally occurring phenolic compounds found in various plants such as red grapes, grape skins and seeds, berries, peanuts, and red wine [1,2]. This compound has garnered significant attention due to its important biological activities [3,4], including anticancer [5,6], antioxidant [7], cardioprotective [8], anti-obesity [9,10], and anti-inflammatory [11,12] properties. Despite its promising therapeutic potential, the clinical use of RSV is limited by its poor aqueous solubility (<0.05 mg/mL), which results in very low bioavailability [13].

It is estimated that the problem of low solubility, and consequently low bioavailability, affects approximately 70–90% of new drug candidates [14]. Substances in the amorphous or nanocrystalline state generally exhibit better solubility compared to crystalline forms. However, the use of amorphous substances is often associated with challenges related to their low thermodynamic stability. One approach to stabilize them is through intimate mixing with a polymer to form an amorphous solid dispersion (ASD) [15,16,17]. Such a sample is homogeneous on a spatial scale, with drug and polymer domains having average dimensions smaller than 100 Å.

The stability of such a system is influenced by many factors, including the type of drug and polymer, their proportions, and the interactions between the active pharmaceutical ingredient (API) and external factors, such as the time since preparation and storage conditions (temperature and humidity) [14]. Specific interactions play a crucial role in the formation of ASDs, with hydrogen bonds being particularly important. These bonds can form between API or nutraceutical molecules and the polymer matrix, stabilizing the ASD, or between drug molecules, which may promote the formation of the crystalline phase (recrystallization) of the substances. However, even if hydrogen bonds are formed between the drug and polymer, too high a drug content in the ASD may promote crystallization. Furthermore, exposing the ASD to significant humidity can lead to polymer plasticization [18] and may also encourage drug crystallization over time. Therefore, studying the stability of various APIs, prepared and stored under different conditions, is important both from a basic and practical perspective.

Additionally, the reduction in crystal size in the nanocrystallization process leads to an increase in solubility and the dissolution rate, which are associated with an increase in the surface-to-volume ratio (s/v) and a decrease in surface energy [19,20]. Nanocrystals can be obtained by reducing the size or crushing the crystals through milling or high-pressure homogenization (the so-called top-down method). Another approach to obtain API nanocrystals of specific sizes entails cultivation in limited volumes (the bottom-up method). Nanocrystallization in porous materials [21,22,23,24], such as porous aluminum silicas, controlled pore glasses (CPGs), zeolites, or polymeric materials, plays a particularly important role in this process. These materials act as soft or hard confinement, where their pore sizes play a critical role in determining the final size and morphology of the resulting nanocrystals. The confinement effect of the pores restricts excessive crystal growth, enabling the production of uniformly sized nanocrystals. However, it is important to note that if the pores are too small, crystal formation may be entirely inhibited due to spatial limitations that prevent proper molecular arrangement [21,22]. For instance, nanocrystalline fenofibrate was formed in pore sizes greater than 20 nm [22]. Also, the type of polymorph can depend on the size of the pores [25]. As discussed, confinement within porous materials not only regulates nanocrystal growth but also contributes to enhancing key physicochemical properties. By limiting particle size and stabilizing the nanocrystal structure, such confinement can enhance solubility, improve the dissolution rate, and increase the physical and chemical stability of the drug. These advantages make confined nanocrystallization particularly attractive for pharmaceutical applications, especially for the formulation of poorly soluble or unstable drugs where enhanced bioavailability is crucial.

Many works [15,17,26] have been devoted to the study of the physical stability of the systems discussed above, increasing the solubility of the incorporated substance, and understanding recrystallization processes and/or crystal growth. However, much less work concerns the characterization of molecular motions, especially in a wide temperature range.

**Figure 1:**
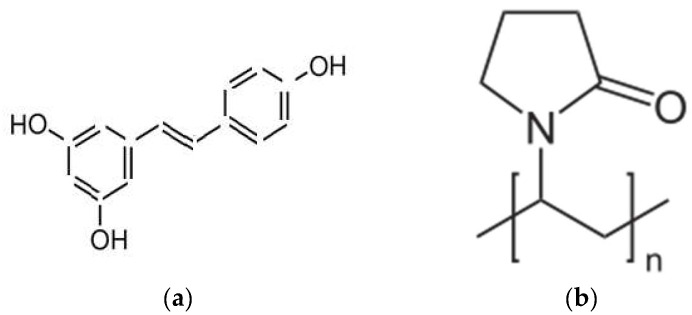
Sketches showing the chemical structures of (**a**) a resveratrol molecule and (**b**) a PVP monomer.

Resveratrol (Figure 1a) crystallizes in the monoclinic system, and the nearly flat molecules with three –OH (hydroxyl) groups are interconnected by a three-dimensional network of strong hydrogen bonds [27,28], which results in a high melting point of 262 °C. It was shown that the amorphous form of resveratrol cannot be obtained by methods such as rapid solvent evaporation, rotary evaporation, or cryomilling of the crystalline material, and therefore it can be considered a rapid crystallizer [29]. However, the formation of an ASD with polymers effectively prevents the crystallization of RSV, which forms ASDs with the following polymers: polyvinylpyrrolidone (PVP), Eudragit E100 (E100), hydroxypropyl methylcellulose (HPMC), hydroxypropyl methylcellulose acetate succinate (HPMCAS), carboxymethyl cellulose acetate butyrate, and polyacrylic acid (PAA) [29]. Each of these polymers inhibits or hinders RSV recrystallization to varying degrees, provided that the proportion of RSV does not exceed a specific value, which also depends on the preparation method of the system. A stable ASD (over time at high humidity) is RSV with PVP, as long as the proportion of RSV does not exceed 45%. When this limit is exceeded, crystalline RSV is observed.

This study aims to investigate the physical stability and molecular mobility of RSV incorporated into a polymeric matrix of PVP (Figure 1b). By combining powder X-ray diffraction (PXRD) and time-domain nuclear magnetic resonance (TD-NMR) methods, we gain insight into the molecular dynamics and the size of RSV crystallites in polymer-encapsulated forms, with a focus on how factors such as humidity and temperature influence these properties. The use of TD-NMR, which has been previously demonstrated for studying ASD systems like felodipine/PVP [30], offers a novel approach to understanding the stability of different systems under varying environmental conditions. In this study, we prepared two PVP/RSV samples with different RSV contents (30% and 50%) and assessed their stability over time and humidity conditions. Additionally, measurements of the ^1^H T_1_ spin–lattice relaxation time in a wide temperature range (performed rarely for ASD systems) give exceptional information about molecular dynamics. Molecular dynamics (MD) simulations, as a powerful tool for exploring ordering behaviors and characterizing the hydrogen bond network [31,32], were also conducted for the PVP/RSV samples.

## 2. Results and Discussion

We prepared two samples, with two different weight contents of RSV incorporated in a PVP matrix: 30% (PVP70/RSV30) and 50% (PVP50/RSV50). After preparation, the material was divided into two parts and stored in different conditions—one at 27 °C and 0% RH (relative humidity), and another at 27 °C and 75% RH. For details, see Section 3. Powder X-ray diffraction and time-domain NMR measurements were performed as a function of the time since preparation of the samples. Additionally, time-domain NMR data as a function of temperature were collected for the samples stored in 0% RH.

### 2.1. Effectiveness of the Preparation of Amorphous Solid Dispersion Systems

**Figure 2:**
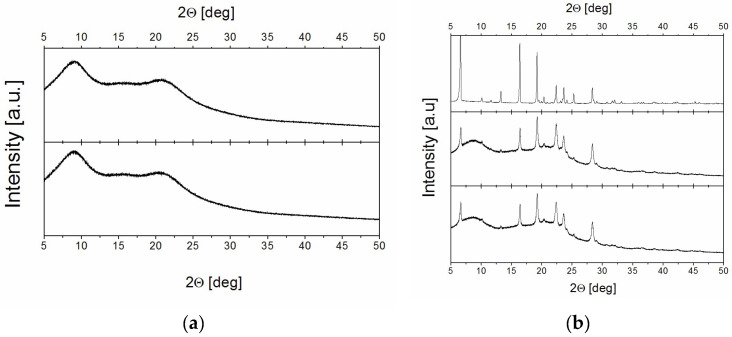
PXRD spectra for (**a**) bottom panel: PVP70/RSV30 in 0% RH just after preparation, top panel: PVP70/RSV30 stored in 75% RH for 300 days; (**b**) bottom panel: PVP50/RSV50 in 0% RH just after preparation, middle panel: PVP50/RSV50 stored in 75% RH for 300 days, top panel: crystalline resveratrol.

First, powder diffraction measurements were performed for the studied samples (Figure 2). For PVP70/RSV30, just after preparation, no diffraction peaks were observed, only a broad halo with two maxima at 8° and 21° (2Θ), confirming the amorphous state of the sample (Figure 2a). PXRD measurements were repeated for this sample after 300 days of storage in 0% RH and no changes were noticed. Similar measurements were taken for this sample immediately after being placed in 75% RH and after being stored for 300 days in this humidity. Similarly, we only observed broad peaks indicating the amorphous nature of the sample and showing that it had not changed over time.

The PXRD spectrum of PVP50/RSV50 was quite different—apart from the wide halo, at least six distinct and well-formed diffraction peaks were observed, with their positions corresponding to those of crystalline RSV (Figure 2b). Figure 2b shows the spectrum immediately after sample preparation and after 300 days of storage in 75% RH. It should be noted that the PXRD spectrum for the sample stored for 300 days in 0% RH is identical to that recorded for the just-prepared sample. Analysis of the PXRD spectrum revealed that the PVP50/RSV50 sample is inhomogeneous, containing both an amorphous phase and crystalline RSV. These findings agree qualitatively with the results reported in [29].

A more complete and precise characterization of these systems is provided by relaxometry NMR (time-domain NMR), as discussed in detail in [30,33] and with basic equations in the Supplementary Information (SI). For PVP70/RSV30, the magnetization recovery curve was well-fitted by a one-exponential function (Equation (S1), Figure S1a), and the determined value of the ^1^H NMR T_1_ relaxation time was 350 ms (±5%). This indicates that the sample is a one-phase (homogeneous) system, which aligns with the diffraction results. In contrast, the magnetization recovery for the PVP50/RSV50 sample exhibited a bi-exponential behavior (Equation (S2), Figure S1b), and the T_1_ times determined were 360 ms and 1100 ms. The non-exponential magnetization recovery strongly suggests that the system is non-homogeneous and consists of two distinct phases.

Using Equation (S3), we determined the fraction of each phase to be 65% (shorter T_1_ component) and 35% (longer T_1_ component), respectively. As the amplitude of magnetization is proportional to the number of protons in each phase [34], it was possible to assign the two components based on the known composition of the sample, specifically the number of protons associated with PVP and RSV. The proportion of protons belonging to RSV molecules in relation to the total number of protons in the sample was 37%, which closely matches the fraction recorded for the longer T_1_ component. Therefore, we can assign this component to nearly all the RSV present in the system, while the shorter component corresponds to PVP with a small amount of RSV. This result indicates that the PVP50/RSV50 system contains a fraction of resveratrol that is not bound to PVP or not dispersed within it. Based on the PXRD studies, we can confirm that this component is crystalline and is likely formed during the system’s preparation. Our observations support the conclusion that resveratrol is a rapid crystallizer [29]. A detailed analysis of the crystals will be presented later in this work.

In conclusion, we can state that at lower concentrations of resveratrol, PVP effectively prevents crystallization, and we have an amorphous solid dispersion system. However, at higher concentrations (e.g., 50%), the system’s ability to stabilize the amorphous state diminishes, leading to crystallite formation.

### 2.2. Systems Stability in 0% and 75% RH

An important question is the physical stability of the prepared systems at different humidity levels. As we have already demonstrated, time-domain NMR is an effective method for monitoring system stability [30]. Therefore, for both samples, relaxation time T_1_ measurements were performed as a function of the time since preparation under two different humidity conditions (0% and 75% RH). Measurements were conducted over a period of 300 days, with intervals of approximately one week.

#### 2.2.1. 0% Relative Humidity

**Figure 3:**
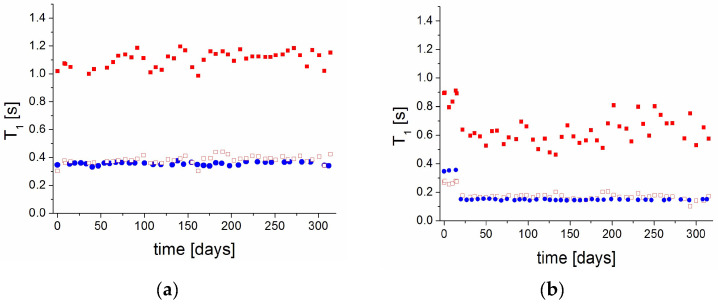
Spin–lattice T_1_ relaxation times as a function of the time since the sample preparation for samples (**a**) stored in 0% RH and (**b**) in 75% RH; ● PVP70/RSV30, □ short component,  ■ long component of PVP50/RSV50.

The T_1_ measurements for the PVP70/RSV30 sample showed a constant value of 350 ms throughout the entire observation period (Figure 3a), with one-exponential magnetization recovery. This indicates that the system remained stable over time. Similar measurements were performed for the PVP50/RSV50 sample. In this case, the magnetization recovery curves were consistently fitted by two exponential functions, yielding two stable T_1_ values throughout the entire period (Figure 3a). The magnetization fractions also remained unchanged, indicating system stability and the continued stability of RSV crystallites.

After the 300-day period, PXRD measurements were repeated for both samples. The results confirmed the amorphous nature of the PVP70/RSV30 system and the presence of crystallites in PVP50/RSV50 (Figure 2).

#### 2.2.2. 75% Relative Humidity

Again, the PVP70/RSV30 sample exhibited a one-exponential magnetization recovery throughout the study. However, after one week of storage in 75% RH, the T_1_ relaxation time decreased to 150 ms, likely due to the incorporation of water into the matrix, and remained at this value for the following 300 days until the end of the measurement period (Figure 3b). The one-exponential recovery indicates that the system remained as a homogeneous single phase. Additionally, the reduction in the T_1_ value suggests increased molecular mobility in the system. In the case of the PVP50/RSV50 sample, the magnetization recovery remained bi-exponential throughout the entire period. After two weeks, the T_1_ values decreased to 170 ms and 600 ms for the shorter and longer components, respectively. Notably, the fractions of the magnetization components also changed. The magnetization fraction assigned to PVP increased to 74%, while that assigned to crystalline resveratrol decreased to 26%, and these values remained stable over time. The increase in PVP-related magnetization suggests the incorporation of water molecules into the polymer matrix.

After the 300-day period, PXRD measurements were repeated for both samples. The results again confirmed the amorphous nature of the PVP70/RSV30 system and the presence of crystallites in PVP50/RSV50 (Figure 2).

Both samples demonstrated stability over time, for at least 300 days from their preparation, in both 0% and 75% relative humidity. This behavior contrasts with that of ASDs of felodipine with PVP (prepared in 50/50 proportions), which are amorphous and stable in 0% RH over time, but undergo a gradual recrystallization of felodipine when stored in 75% RH [30,35]. For RSV, no such recrystallization process was observed in this work. This may be attributed to the highly developed network of hydrogen bonds, which potentially counteracts recrystallization, as already suggested in Ref. [36]. Other work also shows that systems with highly developed networks of hydrogen bonds are stable.

#### 2.2.3. The Analysis of the Phase Composition in 0% and 75% RH

The analysis of the phase composition was made on the basis of the ^1^H NMR spin–spin T_2_ relaxation time, including the analysis of the shape of the free induction decay (FID) curves. This method is a valuable tool for such kinds of analyses [18,37].

**Figure 4:**
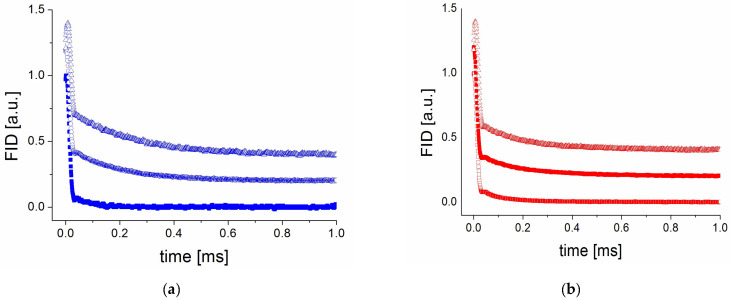
FID curves measured for the (**a**) PVP70/RSV30 and (**b**) PVP50/ASD50 samples on the first day since preparation (0% RH; bottom line), just after placing the samples in 75% RH (middle line), and after 300 days in 75% RH (top line).

For the PVP70/RSV30 and PVP50/RSV50 samples, the FID signal was recorded immediately after preparation and monitored as a function of time for both humidity levels. The observed FID signal for both samples (Figure 4), in 0% RH and 75% RH, exhibited two components and can be well described by the sum of a Gaussian function and an exponential function (Equation (S4)).

The component described by the Gaussian function (with a shorter T_2_^G^ value) corresponds to the rigid part of the studied system. The spin–spin relaxation time (T_2_^G^) for this component was 10 µs for both samples and remained unchanged over time following preparation. The fraction (Equation (S5a)) of this component in the samples immediately after preparation was approximately 90%.

In contrast, the component described by the exponential function indicates the presence of a small, more mobile component. In 0% RH, the spin–spin T_2_^E^ relaxation time for this component remained constant at 80 µs and 95 µs during 300 days for the PVP70/RSV30 and PVP50/RSV50 samples, respectively. Also, its fraction remained stable over time. The situation is different in 75% RH, where for both samples, immediately after exposure we observed changes in the shape of the FID curves and the width of the second component increased significantly (up to T_2_^E^ equal 200 µs), and its fraction (Equation (S5b)) increased to 25% and 15% for PVP70/RSV30 and PVP50/RSV50, respectively. This indicates that water molecules were incorporated into the polymer–resveratrol system and caused their plasticization.

The fraction of this component remained stable for PVP50/RSV50, in contrast to PVP70/RSV30, in which the fraction of this component increased to 40% after 300 days. The increasing fraction additionally suggests that water molecules slowly incorporate into the polymer–resveratrol system, increasing its plasticization (increasing the disordered component) over time.

### 2.3. The Relaxation Processes: The Spin–Lattice Relaxation Time as a Function of Temperature

In order to obtain precise information about the molecular motions in the PVP70/RSV30 and PVP50/RSV50 samples in 0% RH, T_1_ relaxation time measurements were performed over a temperature range going from room temperature to 80 K during both cooling and heating. The measured T_1_ values during heating were identical to those obtained during cooling, indicating no hysteresis. Additionally, measurements for pure PVP to be used as a reference were also performed.

(a) PVP

**Figure 5:**
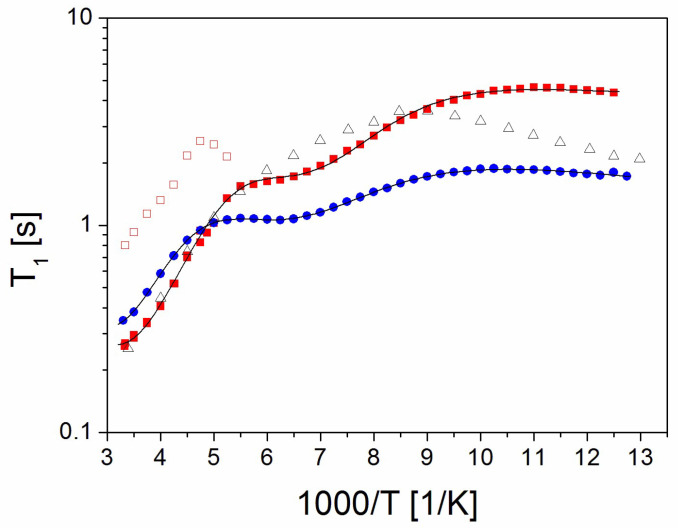
^1^H spin–lattice relaxation time T_1_ values for (Δ) PVP, (●) PVP70/RSV30, and the (■) (□) PVP50/RSV50 shorter and longer components. The solid lines are the best fit to experimental points of Equation (1) including 3 relaxation processes.

The T_1_ (1000/T) dependence (Figure 5) for PVP within the studied temperature range exhibits two relaxation processes: high-temperature visible in temperature range from 300 K to 111 K and low-temperature visible below 111 K related to the decreasing of T_1_ with lowering temperature. The latter is related to the motions of the PVP side chains, while the high-temperature process is attributed to relaxation driven by the motions of the PVP main chain. The activation energy can be estimated from the slope T_1_ (1000/T); for the high-temperature process it is 8.5 kJ/mol, while for the low-temperature process it is much lower at 2.1 kJ/mol (Table 1). A comparison between our results and those reported in the literature for lyophilized (water-free) PVP [38] (measured at the same resonance frequency of 25 MHz) reveals a similar temperature dependence of T_1_ and a comparable maximum value. The fundamental difference between these results is the significant shift of the temperature dependence of T_1_ for lyophilized PVP towards higher temperatures. This shift demonstrates that hydrated PVP is a much more mobile system due to the loosening effect of water molecules.

(b) PVP70/RSV30 and PVP50/RSV50 Samples

Throughout the entire temperature range, the magnetization recovery of PVP70/RSV30 follows a one-exponential behavior, indicating that the sample remains a one-phase system. On the other hand, the magnetization recovery curves of PVP50/RSV50 are bi-exponential from room temperature down to 190 K, allowing the determination of two T_1_ relaxation times (Figure 5). Following the previous analysis, the magnetization component with a long T_1_ relaxation time is attributed to crystalline RSV, while the component with a short T_1_ relaxation time is associated with PVP containing a small number of RSV molecules. When the sample is cooled down to 222 K, both T_1_ relaxation times increase. Next, the value of T_1_ for the longer component starts to decrease, and at 182 K it reaches the same value as the component with a short T_1_ relaxation time, and both have comparable T_1_ values. At lower temperatures, the magnetization recovery curve becomes one-exponential, corresponding to a single T_1_ value.

By comparing the T_1_ dependence for both samples, we can say that they basically show a similar behavior. In the range from 300 K to 200 K, the relaxation time increases by about 160 K, and we observe a weakly developed minimum, while below this temperature T_1_ slightly decreases. The change of the slope of T_1_ in this temperature range (111 K–80 K) is definitely smaller than that of PVP. We can say that this reorientation process shifts towards lower temperatures, which may suggest that in the case of PVP70/RSV30 and PVP50/RSV50 systems, the side motion is faster than in PVP. We can therefore distinguish three relaxation processes, which can be fitted by using the classic BPP formula [39]:

(1) 1T1=C⋅τc1+ωoτc22+4τc1+4ωoτc22,

where C is the relaxation constant, ω_0_ stands for the Larmor frequency, and τ_c_ is the correlation time described by the well-known Arrhenius relation τ_c_ = τ_0_exp(E_A_/RT), where E_A_ denotes the activation energy and τ_0_ is the pre-exponential factor.

Fitting Equation (1) allowed us to determine the activation energies of the high-temperature process and the activation parameters for the minimum T_1_ (intermediate process). The higher activation energies for the PVP70/RSV30 and PVP50/RSV50 systems than for PVP with the HT process indicate that the side motions of the polymer chain are slower and the system is stiffer.

The relaxation process reflected by the T_1_ minimum is also interesting. As it is not observed for PVP, this process should be related to the motions/reorientation of RSV molecules. This motion is more effective for PVP70/RSV30, confirming that in the PVP50/RSV50 system, only part of the RSV is incorporated into the PVP matrix, and crystalline resveratrol occurs in this system.

To summarize, temperature measurements of T_1_ showed the occurrence of reorientation of resveratrol molecules or their fragments (rings) in the PVP70/RSV30 and PVP50/RSV50 samples. It should be remembered that in the crystal, the molecules are bound together by a network of hydrogen bonds (three per molecule) and therefore do not show reorientation in a wide temperature range (they are rigid on the NMR time scale). Mixing resveratrol with PVP causes a significant portion of the molecules to bind to each other or to the polymer by only one or two hydrogen bonds (see discussion in Section 2.5). This results in an increase in the rotational degrees of freedom of the resveratrol molecules. Reorientation of resveratrol molecules is particularly effective in modifying the T_1_ (1000/T) relationship for the PVP70/RSV30 sample because all the resveratrol contained in it is in an amorphous form. However, for the PVP50/RSV50 sample, the effect of resveratrol molecule reorientation on T_1_ (1000/T) is smaller because part of the resveratrol is in the form of nanocrystals. Temperature measurements of T_1_ time also revealed that the presence of resveratrol (both amorphous and nanocrystalline) modifies the molecular dynamics of PVP. It increases the barriers to the reorientation of the PVP backbone, which is caused by steric hindrances induced by resveratrol.

### 2.4. Characterization of Resveratrol Nanocrystals in PVP50/RSV50 (RH 0%)

Since both the PXRD measurements and the magnetization recovery curves of PVP50/RSV50 indicate the presence of crystalline RSV, we tried to determine the size of these crystals from the widths of the diffraction peaks using the Scherrer method [40,41]. Calculations were performed for four peaks (6.6°, 16.4°, 19.2°, and 22.4° (2θ)), and the average crystallite size obtained on their basis was 40 nm. PXRD measurements were also performed for PVP50/RSV50 stored under 75% RH. It turned out that the widths of the diffraction peaks remained the same. Therefore, we conclude that humidity does not affect the size of RSV crystallites. Similar calculations were performed for pure crystalline RSV. Comparison of the PXRD spectra for pure RSV and PVP50/RSV50 showed that (1) as mentioned above, the peak positions are the same for both samples, and (2) the peaks for the former are much narrower. The estimated size of the crystallites in pure RSV based on the peak widths was 65 nm.

It is also worth considering whether RSV nanocrystallites in the PVP50/RSV50 sample occur individually or as aggregates/grains, similar to pure RSV. If they occur individually, this system would represent an interesting example of RSV nanocrystals embedded in a polymer matrix. Although nanocrystallization processes are widely studied both in porous systems and polymers [21], to our knowledge, the sizes of RSV crystallites in PVP have not been previously characterized.

As mentioned earlier, direct evidence for the presence of RSV crystallites in this sample, in addition to PXRD spectra, is provided by the bi-exponential magnetization recovery. In a non-homogeneous system composed of two or more subsystems with different T_1_ relaxation times, if at least one subsystem has a size smaller than a specific parameter A, the magnetization recovery will exhibit one-exponential behavior due to spin diffusion [42,43]. Conversely, if the dimensions of both subsystems are larger than A, the magnetization will follow a bi-exponential function. Therefore, the analysis of the magnetization behavior in such non-uniform systems can be a valuable method for assessing the dimensions of the subsystems. Therefore, the parameter A can be treated as the size of the crystallites, and its value can be determined from the formula [44,45] below:

(2) $$A=\pi \sqrt{\frac{D_{S}\;T_{1}^{1}\;T_{1}^{2}}{\sqrt{8}\left|T_{1}^{2}-T_{1}^{1}\right|}}$$

where D_s_ is the spin diffusion coefficient and T_1_^1^ and T_1_^2^ are the relaxation time values for subsystems 1 and 2, respectively.

Assuming a diffusion coefficient (D_s_) for RSV [46] of 0.8 nm^2^/ms and the T_1_ relaxation times at room temperature determined in this study—T_1_^1^ = 0.36 s for crystalline RSV and T_1_^2^ = 1.1 s for PVP—an A value of 43 nm was obtained. This indicates that the observation of bi-exponential magnetization recovery suggests the presence of crystallites larger than approximately 43 nm in the system. Notably, this value is very close to the crystallite size determined from the diffraction peak widths. To the best of our knowledge, this is the first determination of the size of RSV nanocrystals in a PVP matrix.

### 2.5. Intermolecular Interactions and Molecular Mobility–Molecular Dynamics Simulations Study

**Figure 6:**
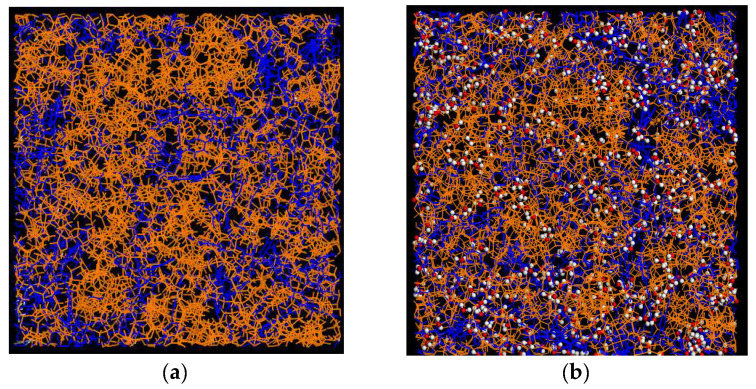
Snapshot of a supercell of ASD (**a**) PVP70/RSV30 (**b**) and PVP70/RSV30/H_2_O systems corresponding to the final configuration after the minimization procedure. PVP chains are represented in orange, RSV—blue, water: oxygen—red, hydrogen—white.

First, we focused on analyzing the local structure of the polymer and RSV within the ASDs. Figure 6 presents snapshots of the amorphous boxes of ASDs formed by PVP/RSV and PVP/RSV/H_2_O following the minimization procedure.

#### 2.5.1. Intermolecular Interactions

Next, the pair distribution functions (PDFs or g(r)), based on the MD simulations, were calculated for both systems. In general, g(r) measures the probability of finding any atom at a distance r from a reference atom, taken as the origin of the coordinates, and can be calculated using the following equation:

(3) $$g\left(r\right)=\frac{1}{4\pi \rho_{0}r^{2}N}\sum_{i}\sum_{j\neq i}\delta \left(r-r_{ij}\right)$$

where *ρ*_0_ is the average number density of atoms, N is the total number of atoms, and r_ij_ is the distance between atoms *i* and *j*.

**Figure 7:**
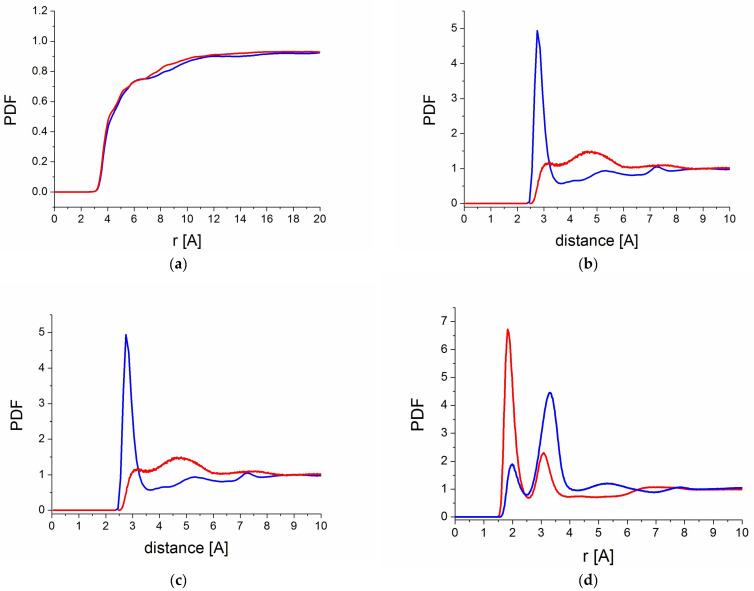
Intermolecular pair distribution functions (**a**) between oxygens for all atoms, (**b**) between oxygens in RSV, (**c**) between oxygens from –C=O groups of PVP chains and hydrogens in RSV, and (**d**) between oxygens from –C=O groups of PVP chains and hydrogens in water molecules. Blue lines—PVP70/RSV30, red lines—PVP70/RSV30/H_2_O systems.

Figure 7a shows the intermolecular part of the PDF confirming the amorphous structure of all of them. To explore the nature of the intermolecular interactions in more detail, and in particular the role and extent of H-bonding, we analyzed the partial PDF corresponding to interactions between RSV molecules, between the PVP chain and RSV, and additionally, for the PVP/RSV/ H_2_O system, the interactions of PVP and RSV with water.

Figure 7b shows the partial PDF corresponding to O-O interactions between RSV molecules. For the RSV in the PVP/RSV30 system, we observe a narrow, intense peak at approximately ~2.725 Å, typical of H-bonded systems, highlighting the significant role of H-bonding in determining the local arrangement of RSV molecules. The situation is completely different for the system with water, where the first peak is observed at around 3 Å, but its intensity is considerably smaller. This shows that the introduction of water to the system practically eliminates hydrogen bonds between RSV molecules.

A similar behavior is observed in the interactions of RSV with PVP. For the PVP/RSV30 system, clear peaks at 2 Å and 3 Å indicate hydrogen bonding of RSV with the polymer chain (Figure 7c). However, the addition of water leads to an almost complete disappearance of both peaks, indicating a very strong reduction in the number of hydrogen bonds between RSV and PVP. The reason for this is that both RSV and PVP exhibit a preference to form H-bonds with water instead of each other, as shown in Figure 7d.

#### 2.5.2. Hydrogen Bond Analysis

Building on the insights provided by the partial PDFs, we quantified the numbers and types of hydrogen bonds present in the different systems. Hydrogen bonds were identified based on the geometrical criteria of a donor–acceptor distance shorter than 3.5 Å and an acceptor–donor–hydrogen angle below 30 degrees [47,48]. The formation of hydrogen bonds in ASDs has been widely studied, both experimentally and through molecular dynamics simulations [31,32,49,50,51,52,53], because they are one of the key factors affecting crystallization kinetics and/or the stability of amorphous drugs in solid dispersions.

First, we analyzed the hydrogen bonds (HBs) between RSV molecules, since the PDF clearly showed the existence of such bonds. The RSV molecule contains three hydroxyl groups (−OH) that can function as both HB donors and acceptors. When examining these RSV-RSV HBs, we found that a portion of the RSV molecules are not connected—18% in the PVP/RSV30 system—and the number of unconnected RSV molecules drastically increases (up to 78%) in the PVP/RSV/water system, which is consistent with the PDF function (Figure 7b). We also observed that RSV molecules can be connected by a single HB or form dimers and trimers. A significant number of dimers and trimers is found in the PVP/RSV30 system (Table 2). Interestingly, in the system with water, we practically do not observe dimers or trimers between RSV molecules, only single bonds between molecules (~18%).

**Figure 8:**
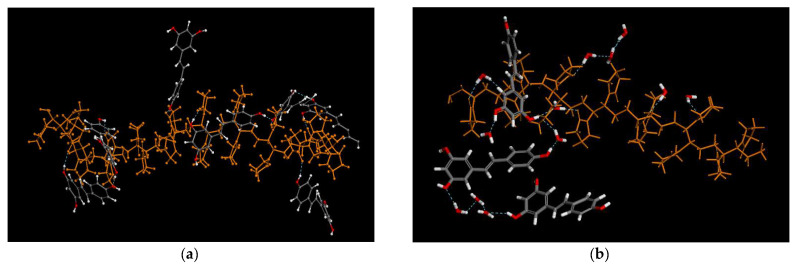
Fragment of (**a**) a PVP chain (orange) with RSV molecules linked to the chain via hydrogen bonding in PVP/RSV30 and (**b**) a PVP chain (orange) with RSV molecules and water molecules linked via hydrogen bonding. In RSV and water molecules: carbon atoms—grey, oxygen—red, hydrogen—white.

It is also interesting to analyze the hydrogen bonds (HBs) between PVP and RSV. Each PVP monomer possesses an amide carbonyl group (−C=O) that can act as an HB acceptor, with a total of 100 acceptors. On average, 27% and 5% of acceptors form hydrogen bonds for PVP70/RSV30 and PVP70/RSV30/H_2_O, respectively. Figure 8 shows a fragment of a polymer chain with RSV molecules connected by hydrogen bonds. It is clearly visible that not all oxygens from the carbonyl groups were used as acceptors.

Analyzing the HBs (Table 2), we can observe that the number of RSV molecules not bound to the polymer also changes—18% for PVP/RSV30—and drastically increases for PVP/RSV/water, where 80% of the molecules are not bound to the PVP chain, and only 18% form one HB with RSV. For the PVP70/RSV30 system, we find that both molecules form one or two bonds with PVP.

The analysis of hydrogen bonds (HBs) between water and resveratrol molecules or with PVP is more complex (Figure 8a). In a water molecule, two hydrogen atoms can be “donated”, while the oxygen atom can act as an acceptor for typically one, two, or even up to three hydrogens donated by neighboring molecules. In the PVP chain, only the carboxyl group can act as an acceptor, while there are no hydrogens to donate. Our results indicate that about 60% of the carboxyl groups in PVP accept either one or two hydrogens from water molecules. Analyzing Table 3, we can also confirm that about 60% of water molecules are bound in hydrogen bonds with the polymer.

However, as mentioned above, the RSV molecule has three hydroxyl groups (−OH) that can act both as HB donors and acceptors. Treating RSV as an acceptor and water as a donor, we find that slightly more than 50% of RSV molecules are not bound to water, 36% form one such bond, and 11% form two bonds. If we analyze the HBs between RSV (treated as a donor), we see that only 3.5% of the molecules are not involved in binding, and more than 96% form one, two, or three hydrogen bonds with water. We see, therefore, that the introduction of water into the system causes the formation of a complex network of hydrogen bonds.

As we can see, in both systems 1 and 2 we find RSV molecules that are not bonded to each other, to the polymer, or to water. Additional analysis showed that we do not find RSV molecules that are not bonded by HBs to any other molecules; in other words, each of the molecules forms at least one HB with RSV, PVP, or water. This means that the hydrogen bond network is very developed and may explain the high stability of both studied systems.

The relation between the strength of hydrogen bonds and the stability of amorphous drugs, as well as the inhibitory effect of several polymers, were studied for felodipine, nifedipine, indomethacin, or acetaminophen dispersed in different polymer matrices [32,54,55,56,57]. Studies showed that stronger and more developed drug–polymer hydrogen bonding correlates with lower molecular mobility and slower crystal growth (recrystallisation) as well as with a better inhibitory effect [58]. Also, molecular dynamics simulations comparing the hydrogen bond network between pure indomethacin and indomethacin in PVP indicate that the network is rebuilt, and more bonds are formed between the drug and the polymer than between indomethacin. It was also shown that the number of hydrogen bonds depends strongly on the drug/polymer concentration ratio [51,59,60], which significantly affects the mobility of the drug molecules. Another recent study revealed the strong relation between the strength of hydrogen bonds and the solubility of the drug [61].

However, introducing water (moisture) into the system may in many cases lead to faster crystallization of amorphous drugs and/or induce an amorphous–amorphous phase separation [62,63]. This may be because water molecules disrupt the network of hydrogen bonds between the drug and the polymer, causing a higher mobility of drug molecules. However, in our case, we did not observe the influence of water on the stability of the PVP70/RSV30/H_2_O sample. MD simulations showed that, on one hand, the numbers of hydrogen bonds between RSV and RSV and between RSV and the polymer decrease. On the other hand, water molecules form HBs with both PVP and RSV, linking RSV molecules and keeping their low mobility (see discussion in the next subsection).

#### 2.5.3. Reorientation Dynamics of RSV in PVP

In this section of the work, we will focus on analyzing the internal mobility and reorientation of RSV incorporated into the polymer matrix. We will consider three torsional angles, the variations in which will provide insights into possible reorientations and/or conformational changes: C4-C5-C7-C8, C5-C7-C8-C9, and C7-C8-C9-C10. Similar to the previous analysis of the dynamic behavior of stilbenoids immersed in the PVP matrix [64], to gain a broader understanding of the geometry of these potential motions, the orientational distribution function P(Θ) was calculated:

(4) $$P\left(\Theta \right)=\frac{1}{N_{step}}\frac{1}{N_{group}}\sum_{i=1}^{N_{step}}N_{i}\left(\Theta ,\Theta +d\Theta \right)$$

where N_i_(Θ,Θ + dΘ) is the number of considered groups found with an orientation in the range (Θ,Θ + dΘ) at step *i*.

**Figure 9:**
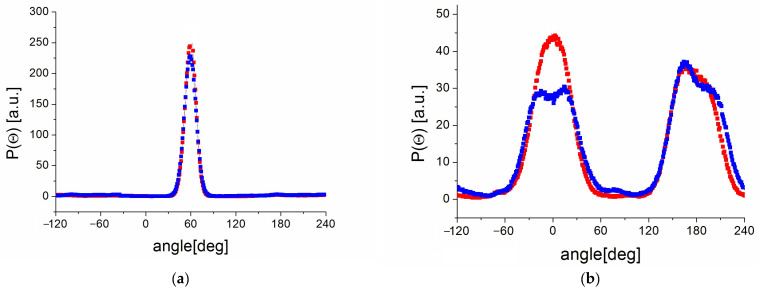
Orientational distribution function of P(Q) for the (**a**) C5-C7-C8-C9 and (**b**) C4-C5-C7-C8 torsional angles for the PVP70/RSV30 (blue points) and PVP70/RSV30/H_2_O (red points) systems.

We can notice (Figure 9a) that the C5-C7-C8-C9 torsional angle is close to 60 degrees, both for the PVP70/RSV30 and PVP70/RSV30/H_2_O systems.

The behavior of P(Θ) for the other two torsional angles (C4-C5-C7-C8 (Figure 9b) and C7-C8-C9-C10) is also interesting—they show two broad maxima at positions 0 and 180 degrees. The analysis of the time dependence of these angles indicates possible reorientations of the rings by large angles. To estimate the time scale of these reorientations, the angular correlation function (ACF) corresponding to the torsional angles considered above was calculated:

(5) $$ACF(t)=\langle {\vec{r}}_{\begin{array}{l} CC \end{array}}\left(t_{0}\right)\cdot {\vec{r}}_{CC}\left(t_{0}+t\right)\rangle$$

where ${\vec{r}}_{CC}\left(t_{0}+t\right)$ is the vector along one C-C bond at time t_0_ + t and the brackets indicate an average for all RSV molecules in the system and possible origins t_0_.

**Figure 10:**
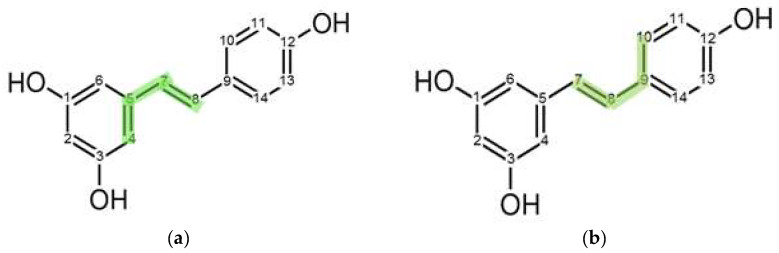

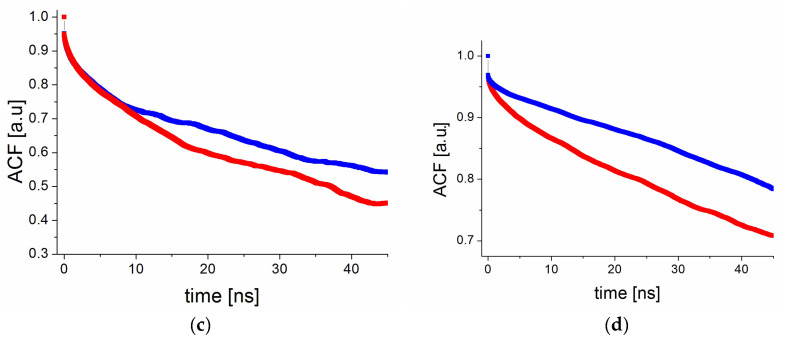
(**a**,**b**) Sketch of the resveratrol molecules showing selected torsional angles (marked in green) and (**c**,**d**) corresponding angular correlation functions for PVP70/RSV30 (blue points) and PVP70/RSV30/H_2_O (red points).

We observe the exponential decay of the AC function for both types of reorientations (Figure 10), which again indicates the existence of RSV motion on the ns timescale. The possibility of such reorientation aligns with the NMR results and the observed T_1_ minimum. We can conclude that the change in the torsional angle C5-C7-C8-C9 is more effective than that in C7-C8-C9-C10. This may be related to the fact that this ring has only one –OH group, meaning the number of possible hydrogen bonds that stiffen the molecule is smaller. Additionally, we see that these reorientations are slightly faster for the PVP70/RSV30/H_2_O system, which indicates that the number of hydrogen bonds influences the mobility of molecules.

## 3. Materials and Methods

*Sample preparation.* Resveratrol (3,4′,5-Trihydroxy-*trans*-stilbene, C_14_H_12_O_3_) was purchased from TCI (TCI R0071), Japan, while polyvinylpyrrolidone (PVP, average molecular weight 10 kg/mol, (C_6_H_9_NO)_n_) was purchased from Sigma-Aldrich (Poznań, Poland) and used without further purification. Figure 1 shows the chemical structure of both compounds.

Samples with two different weight contents of RSV (30% (PVP70/RSV30) and 50% (PVP50/RSV50)) were prepared by the solvent evaporation method described in [29]. Briefly, a mixture of resveratrol and PVP (mass 1700 mg) was dissolved in 250 mL of ethanol. Next, the solution was evaporated using a rotary evaporator. The solvent was removed under vacuum conditions in a rotavapor at 40 °C and 45 rpm. The resultant powder was kept in refrigerator for one day to harden and then was pulverized in a mortar and stored in a desiccator at room temperature for 24 h. The material obtained was divided into two parts. One part of the sample was stored at 27 °C and 0% RH (relative humidity), and the second one was maintained at 27 °C and 75% RH.

*Powder X-ray diffraction.* Powder X-ray diffraction (PXRD) was performed with an EMPYREAN (PANalytical) diffractometer, operating in Bragg–Brentano geometry with CuKα radiation (λ = 1.54 Å), a reflection–transmission spinner (sample stage), and a PIXcel3D detector. Scans were recorded at room temperature (~300 K) at angles ranging from 5 to 50^0^ (2θ) with a continuous scan mode. Data analysis of the size of the crystallites was performed using HighScore Plus software (Version: 3.0e (3.0.5), PANalytical B.V.), which includes a Scherrer Calculator application [41].

*^1^H NMR measurements*. The samples for the ^1^H NMR measurements were placed in a glass ampoule with an inner diameter of 8 mm. All measurements were performed using a relaxometer working at a resonant frequency of 25.0 MHz (El-Lab Tel-atomic). The “solid echo” [65] saturation sequence (25 pulses 90^0^_y_-τ-90^0^_x_ of 4.2 µs) was used and free induction decay (FID) curves were recorded for 64 time intervals ranging from 5 ms to 50 s between the saturation series and the probing pulse. To improve the signal-to-noise ratio (S/N), four accumulations were used. ^1^H spin–lattice T_1_ times were extracted from the amplitude of the FID as a function of τ. ^1^H spin–spin T_2_ relaxation times were calculated from the FID shape.

The ^1^H spin–lattice T_1_ and ^1^H spin–spin T_2_ relaxation times were measured as a function of the time since preparation of the samples approximately every week, up to 300 days. The measurements were performed at a temperature of 295 K. Additionally, for samples stored in 0% RH, T_1_ times were also measured as a function of temperature (during cooling and heating) in the range from room temperature to 80 K.

*Molecular Dynamics.* Molecular Dynamics (MD) simulations were performed on two different systems, corresponding to the samples studied experimentally: PVP70/RSV30 and PVP70/RSV30/H_2_O. The systems were prepared following the procedure described in Ref. [64]. Briefly, the mixed systems were modelled using six PVP chains (each with 100 monomers units) and 120 RSV molecules (PVP70/RSV30), and 120 RSV and 420 water molecules (PVP70/RSV30/H2O) (Table 4). All systems were built using the PACKMOL program [66], initially packing the molecules randomly into a large cubic box with dimensions of ~200 × 200 × 200 Å^3^, corresponding to a density of ~0.05 g/cm^3^. The systems were then simulated at 900 K for about 1 ns using Langevin dynamics in the NVT ensemble at high temperature (900 K). The resulting configurations were then cooled to 260 K at a rate of approximately 6 K/ps. The final densities obtained were about ~1.1 g/cm^3^. This procedure was repeated several times, and the configurations with the smallest total energies were selected as starting points for the production runs. Final trajectories were saved up to 50 ns every 10 ps.

The molecular dynamics (MD) simulations were performed using the LAMMPS package [67]. Intra- and inter-molecular interactions were modeled using the OPLSAA-2005 force field [68], and the TIP4P/2005 model was applied for water molecules [69]. Temperature and pressure were kept constant using a Nosé–Hoover thermostat and barostat with a relaxation constant of 1 ps in both cases [70]. A cutoff distance of 15 Å was applied for the van der Waals forces, and electrostatic interactions were treated using the Particle Mesh Ewald method (particle–particle particle–mesh solver pppm). The nMoldyn [71] program and in-house codes were used to analyze the dynamic and structural properties of the studied systems.

## 4. Conclusions

PXRD measurements, the time-domain ^1^H NMR method, and molecular dynamics simulations lead us to following conclusions:

1. The sample PVP70/RSV30 is a homogenous system, meaning it is an amorphous solid dispersion with resveratrol dispersed in the polymer matrix at the molecular level.
2. The sample PVP50/RSV50 is a system composed of two subsystems, the first of which is crystalline resveratrol and the second of which is a PVP polymer with a small amount of resveratrol molecules connected to it by hydrogen bonds. Crystalline resveratrol was formed already at the stage of its preparation. Resveratrol crystals occur as nanocrystals with sizes about 40 nm, and their sizes were not affected by humidity (75% RH). To our knowledge, this is the first determination of the size of nanocrystals in a PVP matrix, which was performed via two independent methods: Scherrer analysis and NMR spin diffusion constraints.
3. Both samples are stable in 0% RH and 75% RH for at least 300 days from preparation. Humidity influenced mobility and caused plasticization of the studied samples.
4. The incorporation of resveratrol into the polymer matrix makes the molecular dynamics reorientations of these systems more complex compared to pure PVP. In addition to the mobility of the main polymer chains (a process effective at high temperatures with high activation energy) and side chains (a process visible at low temperatures with low activation energy) observed in pure PVP, the reorientations of the resveratrol molecules were also noted, and their activation parameters were determined. Additionally, the mobility of resveratrol molecules was confirmed by molecular dynamics simulations.
5. In the PVP70/RSV30 and PVP70/RSV30/H_2_O samples, the hydrogen bond network is highly developed, which ensures that each resveratrol molecule is linked via at least one hydrogen bond. In the PVP70/RSV30 sample, resveratrol molecules are mainly bonded to other resveratrol molecules, while in PVP70/RSV30/H_2_O they form hydrogen bonds with water molecules. This extensive network of HBs likely contributes to the suppression of recrystallization, even for samples with water.

These findings provide valuable insights into the properties of resveratrol–polymer systems. The comprehensive experimental and computational methods have enabled a detailed understanding of key factors such as the structural properties of the dispersions, the molecular mobility of resveratrol within the polymer matrix, and the nature and strength of hydrogen bonding interactions that contribute to the system’s physical stability and resistance to recrystallization.

## Figures and Tables

**Table 1:** The activation parameters extracted from T_1_ relaxation–spin times measured for PVP, PVP70/RSV30, and PVP50/RSV50 for intermediate- and high-temperature processes.

	Intermediate			High-Temperature
	E_A_ [kJ/mol]	τ_0_ [s]	C [1/s^2^]	E_A_ [kJ/mol]
PVP	-	-	-	8.6
PVP70/RSV30	6.8	2.4 × 10^−11^	5.7 × 10^7^	12.9
PVP50/RSV50	8.7	0.4 × 10^−11^	3.8 × 10^7^	11.9

**Table 2:** Percentage of resveratrol molecules involved in hydrogen bonds.

% Molecules with	HBs RSV-RSV		HBs RSV-PVP	
	PVP70/RSV30	PVP70/RSV30/H_2_O	PVP70/RSV30	PVP70/RSV30/H_2_O
0 HBs	18.0	77.7	18.4	79.1
1 HB	40.1	20.7	38.6	18.3
2 HBs	28.1	1.6	33.3	2.6
3 HBs	11.4	0.1	9.8	0.0

**Table 3:** Percentage of resveratrol molecules involved in hydrogen bonds with water and water molecules with the PVP chain in PVP70/RSV30/H_2_O.

% Molecules with	HBs RSV–Water	HBs RSV–Water	HBs Water–PVP
	RSV (Acceptor)–Water (Donor)	RSV (Donor)–Water (Acceptor)	Water (Donor)–PVP (Acceptor)
0 HBs	51.9	3.5	40.3
1 HB	36.4	23.4	42.7
2 HBs	10.5	42.2	17.0
3 HBs	1.2	30.9	0.0

**Table 4:** Information about the composition, size, and density of the simulated systems.

	PVP70/RSV30	PVP70/RSV30/H_2_O
Number of PVP chains	6	6
Number of resveratrol molecules	120	120
Number of water molecules	-	420
Total number of atoms	13,728	15,078
Size of simulation box after equilibration [Å^3^]	52.04 × 52.04 × 52.04	53.5 × 53.5 × 53.5
Density [g/cm^3^]	1.11	1.11

## Data Availability

The original data presented in the study are available upon reasonable request to the corresponding author.

## Supplementary Materials

The following supporting information can be downloaded at: https://www.mdpi.com/article/10.3390/molecules30091909/s1, Figure S1: Magnetization Mz (points) versus time (τ) distance between the saturating series and probing pulse recorded for (a) PVP70/PVP30 and (b) PVP50/RSV50 samples just after preparation. The lines are the best fit (using Equation (S1) or (S2)) to the experimental points. The resultant magnetization—black, solid lines; blue and green solid lines—two components of the PVP50/RSV50 sample; Figure S2: FID curves measured for PVP70/PVP30 (a,b) 0% RH, 1 day of the study; (c,d) 1 day after placing in 75% RH; (a,c) two components—Equation (S4); blue—Gaussian function; red—exponential function (b,c) experimental points with theoretical line (Equation (S4)); Figure S3: The chemical structure of (a) resveratrol molecule and (b) PVP monomer with types of atoms (blue letters) and partial charges (in e units); Table S1: OPLS-AA2005 parameters for non-bonded interactions; Table S2: OPLS-AA2005 bond parameters; Table S3: OPLS-AA2005 angle parameters; Table S4: OPLS-AA2005 dihedral parameters.

## References

[1] López M., Martínez F., Del Valle C., Orte C., Miró M. (2001). Analysis of phenolic constituents of biological interest in red wines by high-performance liquid chromatography. J. Chromatogr. A.

[2] Tian B., Liu J. (2020). Resveratrol: A review of plant sources, synthesis, stability, modification and food application. J. Sci. Food. Agric..

[3] Frémont L. (2000). Biological effects of resveratrol. Life Sci..

[4] Akinwumi B.C., Bordun K.A.M., Anderson H.D. (2018). Biological activities of stilbenoids. Int. J. Mol. Sci..

[5] Herrera D.P., Chánique A.M., Martínez-Márquez A., Bru-Martínez R., Kourist R., Parra L.P., Schuller A. (2021). Rational design of resveratrol o-methyltransferase for the production of pinostilbene. Int. J. Mol. Sci..

[6] Varoni E.M., Lo Faro A.F., Sharifi-Rad J., Iriti M. (2016). Anticancer molecular mechanisms of resveratrol. Front. Nutr..

[7] Stojanović S., Sprinz H., Brede O. (2021). Efficiency and mechanism of the antioxidant action of trans-resveratrol and its ana-logues in the radical liposome oxidation. Arch. Biochem. Biophys..

[8] Bradamante S., Barenghi L., Piccinini F., Bertelli A.A., de Jonge R., Beemster P., de Jong J.W. (2003). Resveratrol provides late-phase cardioprotection by means of a nitric oxide- and adenosine-mediated mechanism. Eur. J. Pharmacol..

[9] Aguirre L., Fernández-Quintela A., Arias N., Portillo M.P. (2014). Resveratrol: Anti-obesity mechanisms of action. Molecules.

[10] Kim S., Jin Y., Choi Y., Park T. (2011). Resveratrol exerts anti-obesity effects via mechanisms involving down-regulation of adipogenic and inflammatory processes in mice. Biochem. Pharmacol..

[11] Wang K.T., Chen L.G., Tseng S.H., Huang J.S., Hsieh M.S., Wang C.C. (2011). Anti-inflammatory effects of resveratrol and oligostilbenes from Vitis Thunbergii Var. Taiwaniana against lipopolysaccharide-induced arthritis. J. Agric. Food Chem..

[12] Dvorakova M., Landa P. (2017). Anti-inflammatory activity of natural stilbenoids: A review. Pharmacol. Res..

[13] Das S., Lin H.S., Ho P.C., Ng K.Y. (2008). The impact of aqueous solubility and dose on the pharmacokinetic profiles of resveratrol. Pharm. Res..

[14] Nyamba I., Sombié C.B., Yabré M., Zimé-Diawara H., Yaméogo J., Ouédraogo S., Lechanteur A., Semdé R., Evrard B. (2024). Pharmaceutical approaches for enhancing solubility and oral bioavailability of poorly soluble drugs. Eur. J. Pharm. Biopharm..

[15] Pandi P., Bulusu R., Kommineni N., Khan W., Singh M. (2020). Amorphous solid dispersions: An update for preparation, characterization, mechanism on bioavailability, stability, regulatory considerations and marketed products. Int. J. Pharm..

[16] Janssens S., Van den Mooter G. (2009). Review: Physical chemistry of solid dispersions. J. Pharm. Pharmacol..

[17] Kawabata Y., Wada K., Nakatani M., Yamada S., Onoue S. (2011). Formulation design for poorly water-soluble drugs based on biopharmaceutics classification system: Basic approaches and practical applications. Int. J. Pharm..

[18] Litvinov V.M., Penning J.P. (2004). Phase composition and molecular mobility in nylon 6 fibers as studied by proton NMR transverse magnetization relaxation. Macromol. Chem. Phys..

[19] Vollath D., Fischer F.D., Holec D. (2018). Surface energy of nanoparticles—Influence of particle size and structure. Beilstein J. Nanotechnol..

[20] Paredes A.J., McKenna P.E., Ramöller I.K., Naser Y.A., Volpe-Zanutto F., Li M., Abbate M.T.A., Zhao L., Zhang C., Abu-Ershaid J.M. (2021). Microarray patches: Poking a hole in the challenges faced when delivering poorly soluble drugs. Adv. Funct. Mater..

[21] Yang X., Ong T.C., Michaelis V.K., Heng S., Huang J., Griffin R.G., Myerson A.S. (2014). Formation of organic molecular nanocrystals under rigid confinement with analysis by solid state NMR. CrystEngComm.

[22] Dwyer L.M., Michaelis V.K., O’Mahonya M., Griffin R.G., Myerson A.S. (2015). Confined crystallization of fenofibrate in nanoporous silica. CrystEngComm.

[23] Ju S.P., Chang J.G. (2004). A molecular dynamics simulation investigation into the behavior of water molecules inside Au nanotubes of various sizes. Microp. Mesop. Mater..

[24] Lemanowicz M., Mielańczyk A., Walica T., Kotek M., Gierczycki A. (2021). Application of polymers as a tool in crystallization—A review. Polymers.

[25] Rengarajan G.T., Enke D., Steinhart M., Beiner M. (2011). Size-dependent growth of polymorphs in nanopores and Ostwald’s step rule of stages. Phys. Chem. Chem. Phys..

[26] Navnit S., Harpreet S., Choi D., Chokshi H. (2014). Amorphous Solid Dispersions Theory and Practice (Advances in Delivery Science and Technology).

[27] Caruso F., Tanski J., Villegas-Estrada A., Rossi M. (2004). Structural basis for antioxidant activity of trans-resveratrol: Ab initio calculations and crystal and molecular structure. J. Agric. Food Chem..

[28] Zarychta B., Gianopoulos C.G., Pinkerton A.A. (2016). Revised structure of trans-resveratrol: Implications for its proposed antioxidant mechanism. Bioorg. Med. Chem. Lett..

[29] Wegiel L.A., Mauer L.J., Edgar K.J., Taylor L.S. (2013). Crystallization of amorphous solid dispersions of resveratrol during preparation and storage—Impact of different polymers. J. Pharm. Sci..

[30] Pajzderska A., Mielcarek J., Wąsicki J. (2022). The physical stability of felodipine and its recrystallization from an amorphous solid dispersion studied by NMR relaxometry. AAPS PharmSciTech.

[31] Xiang T., Anderson B.D. (2017). Molecular dynamics simulation of amorphous hydroxypropylmethylcellulose and its mixtures with felodipine and water. J Pharm. Sci..

[32] Kothari K., Ragoonanan V., Suryanarayanan R. (2015). The role of drug—polymer hydrogen bonding interactions on the molecular mobility and physical stability of nifedipine solid dispersions. Mol. Pharm..

[33] Pajzderska A., Fojud Z., Jarek M., Wąsicki J. (2019). NMR relaxometry in the investigation of the kinetics of the recrystallization of felodipine. Powder Technol..

[34] Abragam A. (1983). The Principles of Nuclear Magnetism.

[35] Rumondor A.C.F., Stanford L.A., Taylor L.S. (2009). Effects of polymer type and storage relative humidity on the kinetics of felodipine crystallization from amorphous solid dispersions. Pharm. Res..

[36] Wegiel L.A., Mosquera-Giraldo L.I., Mauer L.J., Edgar K.J., Taylor L.S. (2015). Phase behavior of resveratrol solid dispersions upon addition to aqueous media. Pharm. Res..

[37] Litvinov V.M., Cheng H., Asakura T., English A.D. (2011). Molecular mobility and phase composition in polyolefins: From fundamental to applied research. NMR Spectroscopy of Polymers: Innovative Strategies for Complex Macromolecules.

[38] Yoshioka S., Aso Y., Kojima S. (2003). Molecular mobility of lyophilized poly(vinylpyrrolidone) and methylcellulose as determined by the laboratory and rotating frame spin-lattice relaxation times of ^1^H and ^13^C. Chem. Pharm. Bull..

[39] Bloembergen N., Purcell E.M., Pound R.V. (1948). Relaxation effects in nuclear magnetic resonance absorption. Phys. Rev..

[40] Weibel A., Bouchet R., Boulc’H F., Knauth P. (2005). The big problem of small particles: A comparison of methods for determination of particle size in nanocrystalline anatase powders. Chem. Mater..

[41] Muniz F.T., Miranda M.A., Dos Santos C.S., Sasaki J.M. (2016). The Scherrer equation and the dynamical theory of X-ray diffraction. Acta Crystallogr. A.

[42] Meyer H.W., Schneider H., Saalwächter K. (2012). Proton NMR spin-diffusion studies of PS-PB block copolymers at low field: Two- vs. three-phase model and recalibration of spin-diffusion coefficients. Polym. J..

[43] Ernst M., Meier B.H., Ando I., Asakura T. (1998). Studies in physical and theoretical chemistry. Solid State NMR of Polymers.

[44] Qi S., Belton P., Nollenberger K., Clayden N., Reading M., Craig D.Q.M. (2010). Characterisation and prediction of phase separation in hot-melt extruded solid dispersions: A thermal, microscopic and NMR relaxometry study. Pharm. Res..

[45] Belton P.S., Hills B.P. (1987). The effects of diffusive exchange in heterogeneous systems on N.M.R. line shapes and relaxation processes. Mol. Phys..

[46] Clauss J., Schmidt-Rohr K., Spiess H.W. (1993). Determination of domain sizes in heterogeneous polymers by solid-state NMR. Acta Polym..

[47] Luzar A. (2000). Resolving the hydrogen bond dynamics conundrum. J. Chem. Phys..

[48] Prada-Gracia D., Shevchuk R., Rao F. (2013). The quest for self-consistency in hydrogen bond definitions. J. Chem. Phys..

[49] Xiang T., Anderson B.D. (2019). Effects of molecular interactions on miscibility and mobility of ibuprofen in amorphous solid dispersions with various polymers. J. Pharm. Sci..

[50] Zhang N., Chen S., Yang B., Huo J., Zhang X., Bao J., Ruan Z., He G. (2018). Effect of hydrogen-bonding interaction on the arrangement and dynamics of water confined in a polyamide membrane: A molecular dynamics simulation. J. Phys. Chem. B.

[51] Mohapatra S., Samanta S., Kothari K., Mistry P., Suryanarayanan R. (2017). Effect of polymer molecular weight on the crystallization behavior of indomethacin amorphous solid dispersions. Cryst. Growth Des..

[52] Chao J., Li H., Cheng K.W., Yu M.S., Chang R.C.C., Wang M. (2010). Protective effects of pinostilbene, a resveratrol methylated derivative, against 6-hydroxydopamine-induced neurotoxicity in SH-SY5Y cells. J. Nutr. Biochem..

[53] Wang Y., Ma R., Wang B., Liu X., Zhao X., Liu L., Zhang L., Gao Y. (2024). Influence of number and strength of hydrogen bonds on fracture property and microscopic mechanisms of associative hydrogen-bonded polymers via molecular dynamics simulation. Langmuir.

[54] Shi Q., Chen H., Wang Y., Wang R., Xu J., Zhang C. (2022). Amorphous solid dispersions: Role of the polymer and its importance in physical stability and in vitro performance. Pharmaceutics.

[55] Trasi N.S., Taylor L.S. (2012). Effect of polymers on nucleation and crystal growth of amorphous acetaminophen. CrystEngComm.

[56] Kestur U.S., Taylor L.S. (2010). Role of polymer chemistry in influencing crystal growth rates from amorphous felodipine. CrystEngComm.

[57] Taylor L.S., Zografi G. (1997). Spectroscopic characterization of interactions between pvp and indomethacin in amorphous molecular dispersions. Pharm. Res..

[58] Newman A., Zografi G. (2022). What are the important factors that influence api crystallization in miscible amorphous api-excipient mixtures during long-term storage in the glassy state?. Mol. Pharm..

[59] Zhong H., Lu T., Wang R., Ouyang D. (2025). Quantitative analysis of physical stability mechanisms of amorphous solid dispersions by molecular dynamic simulation. AAPS J..

[60] Wilke S.K., Al-Rubkhi A., Benmore C.J., Byrn S.R., Weber R. (2024). Modeling the structure of ketoprofen-poly(vinylpyrrolidone) amorphous solid dispersions with empirical potential structure refinements of x-ray scattering data. Mol. Pharm..

[61] Aulich V., Ludík J., Fulem M., Červinka C. (2024). Molecular insights into kinetic stabilization of amorphous solid dispersion of pharmaceuticals. Phys. Chem. Chem. Phys..

[62] Luebbert C., Wessner M., Sadowski G. (2018). Mutual impact of phase separation/crystallization and water sorption in amorphous solid dispersions. Mol. Pharm..

[63] Hancock B.C., Zografi G. (1994). The relationship between the glass transition temperature and the water content of amorphous pharmaceutical solids. Pharm. Res..

[64] Pajzderska A., Gonzalez M.A. (2023). Molecular dynamics simulations of selected amorphous stilbenoids and their amorphous solid dispersions with poly(vinylpyrrolidone). J. Pharm. Sci..

[65] Powles J.G., Mansfield P. (1962). Double-pulse nuclear-resonance transients in solids. Phys. Lett..

[66] Martínez L., Andrade R., Birgin E.G., Martinez J.M. (2009). Packmol: A package for building initial configurations for molecular dynamics simulations. J. Comput. Chem..

[67] Thompson A.P., Aktulga H.M., Berger R., Bolintineanu D.S., Brown W.M., Crozier P.S., In’t Veld P.J., Kohlmeyer A., Moore S., Nguyen T.D. (2022). LAMMPS—A flexible simulation tool for particle-based materials modeling at the atomic, meso, and continuum scales. Comp. Phys. Comm..

[68] Banks J.L., Beard H.S., Cao Y., Cho A.E., Damm W., Farid R., Felts A.K., Halgren T.A., Mainz D.T., Maple J.R. (2005). Integrated modeling program, applied chemical theory (IMPACT). J. Comput. Chem..

[69] Abascal J.L.F., Vega C. (2005). A general purpose model for the condensed phases of water: TIP4P/2005. J. Chem. Phys..

[70] Allen M.P., Tildesley D. (2002). Computer Simulation of Liquids.

[71] Hinsen K., Pellegrini E., Stachura S., Kneller G.R. (2012). NMoldyn 3: Using task farming for a parallel spectroscopy-oriented analysis of molecular dynamics simulations. J. Comput. Chem..

