# Supplementary material for: Physical Stability and Molecular Mobility of Resveratrol in a Polyvinylpyrrolidone Matrix
Source: Molecules. 2025 Apr 25;30(9):1909. doi: 10.3390/molecules30091909 (PMC12073277; doi:10.3390/molecules30091909)
Supplement: Supplementary file 1 [file molecules-30-01909-s001.zip › molecules-3554905-supplementary.pdf]

Physical stability and molecular mobility of resveratrol in polyvinylpyrrolidone matrix

A. Pajzderska[1], M.A. Gonzalez[2], M. Jarek[3], J. Mielcarek[4], J. Wąsicki[1]*

[1] *A. Mickiewicz University, Faculty of Physics, Uniwersytetu Poznanskiego 2, Poznan, Poland*
[2] *Institute Laue Langevin, 71 Avenue des Martyrs, Grenoble, France*
[3] *NanoBioMedical Centre, A. Mickiewicz University, Wszechnicy Piastowskiej 3, Poznan, Poland*
[4] *University of Medical Sciences, Department of Inorganic and Analytical Chemistry, Poznań Grunwaldzka 6, Poznań, Poland*

The magnetization M_z recovery curves obtained from the evolution of free induction decay (FID) signal for a one-phase system can be expressed as:

$$M_z = M_0\left(1 - \exp\left(-t/T_1\right)\right) \tag{S1}$$

where T_1 represents the relaxation time and M_0 is the total magnetization.

For a two-phase system, where the spin-lattice relaxation times T_1 differ for each phase, the magnetization M_z recovers bi-exponentially, described as:

$$M_z = M_0^1\left(1 - \exp\left(-t/T_1^1\right)\right) + M_0^2\left(1 - \exp\left(-t/T_1^2\right)\right) \tag{S2}$$

where T_1^1 and T_1^2 are the values of the relaxation time in phase 1 and phase 2, respectively; while M_0^1 and M_0^2 represent the contributions to the total magnetization from each phase. The fraction of the each phases can be expressed as:

$$F_{c1} = \frac{M_0^1}{M_o^1 + M_0^2} \tag{S3a}$$

$$F_{c2} = \frac{M_0^2}{M_o^1 + M_0^2} \tag{S3b}$$

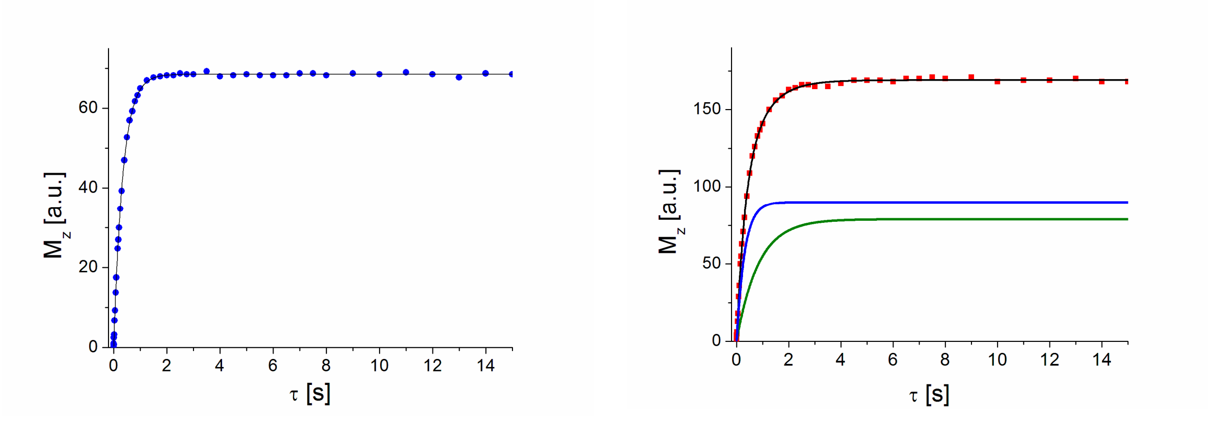

a) b)

Figure. S1. Magnetization M_z (points) versus time (τ) distance between the saturating series and probing pulse recorded for a) PVP70/PVP30, b) PVP50/RSV50 samples just after preparation. The lines are the best fit (using Eq.S1 or S2) to the experimental points. The the resultant magnetization — black, solid lines; blue and green solid lines – two components of PVP50/RSV50 sample

The Free Induction Decay (FID) curves (measured at the maximum value $_{e}$) were fitted using the linear combination of two functions (Gaussian and exponential function):

$$A(t) = A_0^G \, exp(-(t/T_2^G)^2) + A_0^E \, exp\left(-(t/T_2^E)\right) \tag{S4}$$

where A represents the amplitude of each component, and T_2 the spin-spin relaxation time; the superscript “G” indicates the Gaussian function, while superscript “E” refers the exponential component, which is characterised by a longer relaxation time.

The fraction of the each components can be expressed as:

$$F_G = \frac{A_0^G}{A_0^G + A_0^E} \tag{S5a}$$

$$F_E = \frac{A_0^E}{A_0^G + A_0^E} \tag{S5b}$$

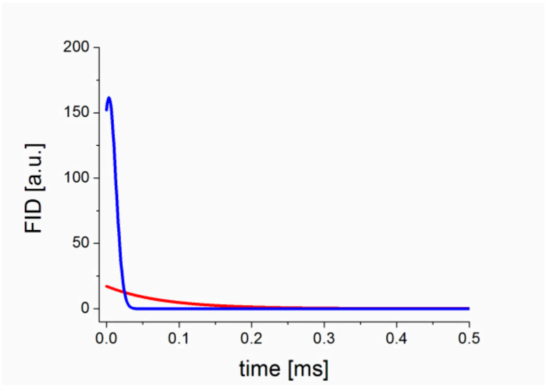

a)

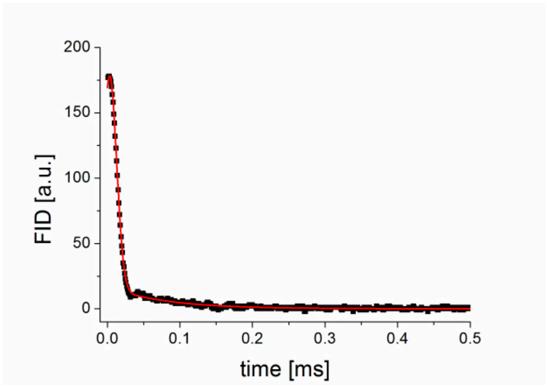

b)

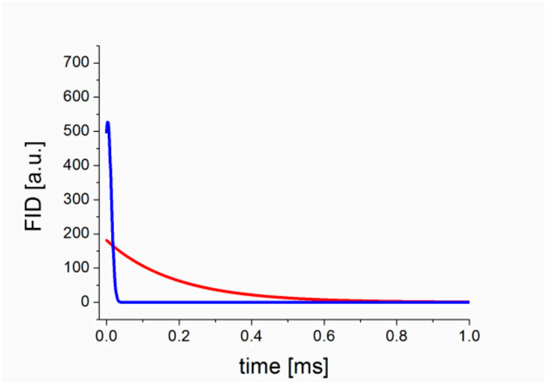

c)

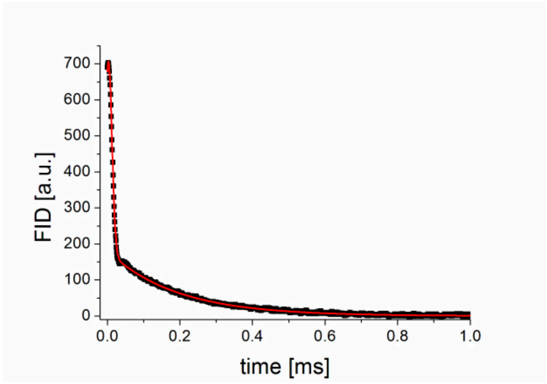

d)

Figure. S2. FID curves measured for PVP70/PVP30 a), b) 0RH, 1 day of the study; c), d) 1 day after placing in 75RH; a), c) two components – eq. S4; blue – Gaussian function; red – exponential function b), c) experimental points with theoretical line (eq. S4)

Form of the force field OPLSAA-2005

Interaction potential is defined by the formula:

$$U(R) = E_{bonded} + E_{non-bonded} \qquad \text{(S6)}$$

Where bonded interactions:

$$E_{bonded} = E_{bond} + E_{angle} + E_{dihedral} \qquad \text{(S6a)}$$

Non-bonded interactions:

$$E_{non-bonded} = E_{LJ} + E_{coul} \qquad \text{(S6b)}$$

Harmonic bond style	
$E_{bond} = k_b (r - r_0)^2$	k_b - the bond-stretching force constant<br>r_0 - the equilibrium bond length
**Harmonic angle style**	
$E_{angle} = k_\Theta (\Theta - \Theta_0)^2$	k_Θ - the angle-bending force constant<br>Θ_0 - the equilibrium value for the bond angle
**Opls dihedral style**	
$E_{dihed} = k_1(1+\cos(\phi)) + k_2(1+\cos(2\phi)) + k_3(1+\cos(3\phi))$	k_1, k_2, k_3 – force constants
**Lennard_Jones potential term**	
$E_{LJ} = 4\varepsilon_{ij}\left[\left(\frac{\sigma_{ij}}{r_{ij}}\right)^{12} - \left(\frac{\sigma_{ij}}{r_{ij}}\right)^{6}\right]$	ε_{ij} - the potential well depth<br>σ_{ij} - the distance where the potential equals zero<br>r_{ij} is the interatomic distance.
**Coulombic term**	
$E_{coul} = \frac{1}{\varepsilon}\frac{q_i q_j}{r_{ij}}$	ε -the dielectric constant<br>q_i and q_j are atomic charges of interacting atoms i and j<br>r_{ij} is the interatomic distance.

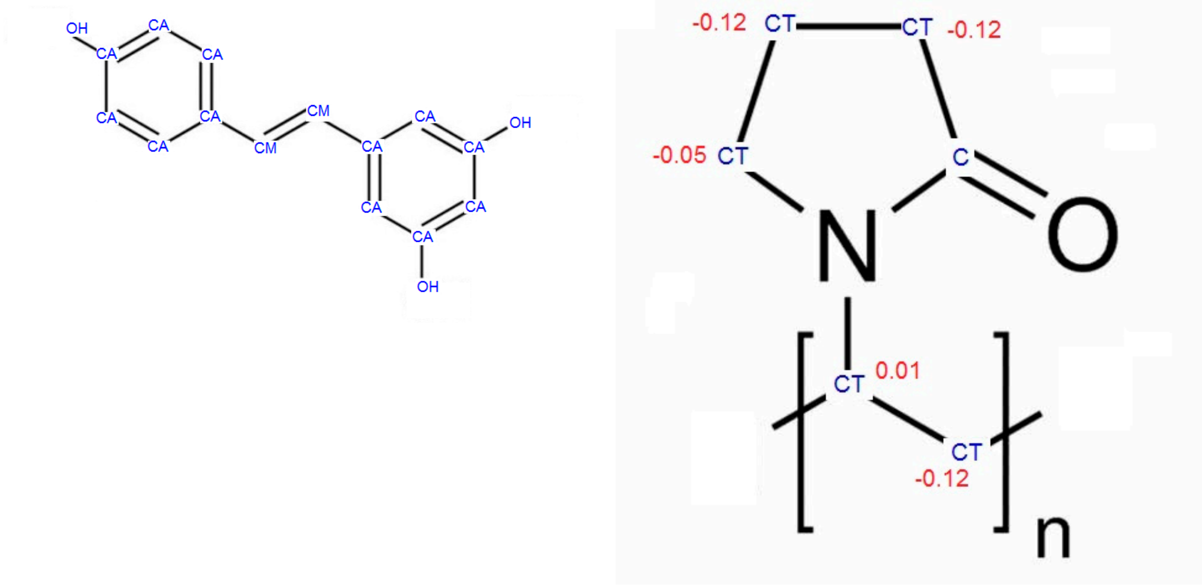

a) b)

Fig. S3. The chemical structure of a) resveratrol molecule, b) PVP monomer with types of atoms (blue letters) and partial charges (in e units).

Table S1. OPLS-AA2005 parameters for non-bonded interactions

Atom	q [e]	ε [kcal/mol]	σ [A]
CA	-0.11500	0.070000	3.550000
CA (attached to OH)	0.15000	0.070000	3.550000
CA (attached to CM)	0.00000	0.070000	3.550000
HA – hydrogen attached to CA	0.11500	0.030000	1.100000
CM	-0.11500	0.076000	3.550000
OH	-0.58500	0.170000	3.070000
HO – hydrogen attached to OH	0.43500	0.030000	0.500000
CT	See Fig. S3	0.066000	3.500000
HC – hydrogen attached to CT	0.06000	0.030000	2.500000
N	-0.14000	0.170000	3.250000
C	0.50000	0.105000	3.750000
O	-0.50000	0.210000	2.960000

Table S2. OPLS-AA2005 bond parameters

Atoms	k_b [kcal/mol]	r_0 [A]
CA - CA	469.0	1.40000
CA - HA	367.0	1.08000
CA - CM	320.0	1.46000
CM - HA	340.0	1.08000
OH -HO	553.0	0.94500
CT - CT	268.0	1.52900
CT - HC	340.0	1.09000
CT - N	337.0	1.44900
C – O	570.0	1.22900
C – N	490.0	1.33500
C - CT	317.0	1.52200

Table S3. OPLS-AA2005 angles parameters

Atoms	k_Θ [kcal/mol/rad^2]	Θ_0 [deg]
CA - CA - CA	63.00000	120.00000
CA - CA - HA	70.00000	120.00000
CA - CM -CA	60.00000	123.66000
OH - CA - CA	70.00000	120.00000
HO-OH-CP	35.00000	113.00000
HA - CM - CA	40.00000	123.66000
HA - CM - HA	35.00000	120.00000
CA - CM -HA	80.00000	117.26300
HC -CT -CT	37.50000	110.70000
HC -CT -N	35.00000	109.50000
CT -CT -N	70.00000	110.72300
CT -CT -CT	58.35000	112.70000
HC -CT -HC	33.00000	107.80000
CT -N -C	50.00000	121.90000
N -C -O	80.00000	122.90000
C -CT -CT	63.00000	111.10000
CT -N -CT	50.00000	118.00000
N -C -CT	70.00000	116.60000
C -CT- HC	35.00000	109.50000
O- C- CT	30.00000	120.40000

Table S4. OPLS-AA2005 dihedral parameters

Atoms	k_1 [kcal/mol]	k_2 [kcal/mol]	k_3 [kcal/mol]
CA - CA - CA - CA	0.00000	7.25000	0.00000
HA - CA - CA - CA	0.00000	7.25000	0.00000
HA - CA - CA - HA	0.00000	7.25000	0.00000
CA - CA - CA - CM	0.00000	7.25000	0.00000
HA - CA - CA - CM	0.00000	7.25000	0.00000
HA - CA - CA - OH	0.00000	7.25000	0.00000
OH - CA - CA - CA	0.00000	7.25000	0.00000
HO - HO - CA - CA	0.00000	7.25000	0.00000
CA - CA - CM - HA	0.00000	0.00000	1.13100
CA - CA - CM - CM	0.31600	3.70700	-0.97400
CA - CM - CM - CA	0.00000	14.0000	0.00000
HA - CM - CM - CA	0.00000	14.0000	0.00000
HA - CM - CM - HA	0.00000	14.0000	0.00000
HC - CT - CT - CT	0.00000	0.00000	0.30000
HC - CT - CT - HC	0.00000	0.00000	0.30000
CT - CT - CT - CT	1.13500	-0.15100	0.40000
N - CT - CT - CT	0.87800	0.98200	0.56800
N - CT - CT - HC	0.00000	0.00000	0.30000
C - CT - CT - CT	-1.91400	-0.36400	0.58900
C - CT - CT - HC	0.00000	0.00000	-0.22500
N - CT - CT - N	4.40100	1.26900	-1.82600
CT - CT - N - C	-0.87900	-0.16200	0.06700
CT - CT - N - CT	3.90000	0.31600	0.11300
CT - N - CT - HC	0.00000	0.00000	0.00000
HC - CT - N - C	0.00000	0.00000	0.00000
CT - N - C - O	0.00000	6.08900	0.00000
CT - N - C - CT	2.99700	3.07900	0.00000
N - C - CT - HC	0.00000	0.00000	0.00000
N - C - CT - CT	2.79500	-0.15900	-0.37500
O - C - CT - HC	0.00000	0.00000	0.00000
O - C - CT - CT	0.00000	0.60300	0.00000
HC - CT - CT - CT	0.00000	0.00000	0.30000
HC - CT - CT - HC	0.00000	0.00000	0.30000
